# *In vivo* imaging of transgenic *Brugia malayi*

**DOI:** 10.1371/journal.pntd.0008182

**Published:** 2020-04-03

**Authors:** Canhui Liu, Sai Lata De, Kristi Miley, Thomas R. Unnasch

**Affiliations:** Center for Global Health Infectious Disease Research, University of South Florida, Florida, United States of America; University of Pennsylvania, UNITED STATES

## Abstract

**Background:**

Studies of the human filarial parasite have been hampered by the fact that they are obligate parasites with long life cycles. In other pathogenic infections, *in vivo* imaging systems (IVIS) have proven extremely useful in studying pathogenesis, tissue tropism and *in vivo* drug efficacy. IVIS requires the use of transgenic parasites expressing a florescent reporter. Developing a method to produce transgenic filarial parasites expressing a florescent reporter would permit IVIS to be applied to the study of tissue tropism and provide a non-invasive way to screen for in vivo drug efficacy against these parasites.

**Methodology/Principal findings:**

We report the development of a dual luciferase reporter construct in a *piggyBac* backbone that may be used to stably transfect *Brugia malayi*, a causative agent of human filariasis. Parasites transfected with this construct were visible in IVIS images obtained from infected gerbils. The signal in these infected animals increased dramatically when the transgenic parasites matured to the adult stage and began to produce transgenic progeny microfilaria. We demonstrate that the IVIS system can be used to develop an effective method for cryopreservation of transgenic parasites, to non-invasively monitor the effect of treatment with anti-filarial drugs, and to rapidly identify transgenic F1 microfilariae.

**Conclusions:**

To our knowledge, this represents the first application of IVIS to the study of a human filarial parasite. This method should prove useful in studies of tissue tropism and as an efficient *in vivo* assay for candidate anti-filarial drugs.

## Author summary

The human filarial parasites are responsible for two major debilitating diseases; lymphatic filariasis (elephantiasis) and onchocerciasis (river blindness). Both have been identified as diseases that can be eliminated as a public health problem. However, the current elimination programs rely upon prolonged distribution of a limited number of drugs, a process which is logistically difficult to accomplish and may encourage the development of resistance. Thus, more effective drugs are needed. In other infectious diseases, *in vivo* imaging systems (IVIS) have proven to be very effective tools to study the pathogenesis of infection and to develop rapid and non-invasive assays for new drugs. Here we report the adaptation of IVIS to a human filarial parasitic infection and demonstrate in principal that it may be used to non-invasively monitor the efficacy of anti-filarial treatment *in vivo*. This system should prove useful as an *in vivo* screen for new anti-filarial compounds, as well as studies of the basic biology of these parasites.

## Introduction

The human filarial parasites are responsible for two major debilitating diseases in the developing world, lymphatic filariasis (elephantiasis) and onchocerciasis (river blindness). Both have been identified as diseases that may be eliminated as a public health problem [1]. The current elimination programs for both diseases rely primarily or exclusively on mass drug treatment programs that use a very limited number of drugs [2–4]. The drugs that are currently used are not only limited in number, but they also must be given over a period of several years to a very high proportion of the at-risk population in order to achieve elimination [5] increasing the chances that resistance may develop. These prolonged mass distribution programs are also quite difficult to accomplish logistically and are very expensive. Thus, there is continued interest in the development of new drugs or vaccines that might eliminate the infection with a single treatment or a small number of treatments given over a short period of time [6].

New drug development for the human filaria has been hampered by the fact that all of these organisms are obligate parasites, and the two most important of them (*Wuchereria bancrofti*, the causative agent of filariasis in Asia, Africa and Latin America and *Onchocerca volvulus*, the causative agent of onchocerciasis in Africa and Latin America) do not have an animal host, infecting only humans. Only *Brugia malayi* (a parasite causing human filariasis in Southeast Asia) can be maintained throughout the full mammalian life cycle in an animal host, most efficiently in the gerbil (*Meriones unguiculatus*) [7–10]. As such, *B*. *malayi* has become an important model for the study of the human filarial infections. However, the biology of *B*. *malayi* infections in gerbils make it a difficult model for evaluating new drugs. The pre-patent period (the time between when infective larvae are introduced into a naïve animal and the time that the fully developed parasites begin to produce progeny) is quite long [11]. And assessing the effect of a drug on the parasite requires sacrificing the infected animal and performing a detailed necroscopy. Thus, a method that could assess the effect of a drug during the pre-patent period and to non-invasively assay for drug efficacy in patently infected animals would accelerate the efforts to develop new anti-filarial compounds.

*In vivo* imaging systems (IVIS) offer a way to monitor the progress of an infection in a non-invasive manner. IVIS has been used to study and evaluate drug and vaccine efficacy in intracellular eukaryotic parasitic [12–14], bacterial [15] and viral infections [16]. IVIS relies on the use of transgenic parasites expressing a florescent reporter protein, whose presence can be detected with a highly sensitive charged coupled device (CCD) camera. Because the process is non-invasive and painless, an individual animal can be imaged repeatedly over a period of time, thus providing a complete picture of the progression of an infection over time.

IVIS requires a florescent reporter be expressed in the pathogen to be imaged, which in turn requires a method to stably transfect the pathogen. Recently, we reported the development of a method to stably transfect *B*. *malayi* molting larvae using the *piggyBac* transposon system [17]. The transgenic parasites were developmentally competent, and the transgenes were stably integrated into the parasite genome and inherited in the subsequent generation [17]. We reported the development of a toolkit of various plasmids based on the *piggyBac* system containing a variety of selectable markers and reporters. Here, we report the construction of another member of this plasmid family, a dual luciferase vector expressing both *Gaussia princeps* and firefly luciferase reporters. We demonstrate that parasites transfected with this construct can be detected in infected animals using IVIS and report a series of studies demonstrating utility of this system. To our knowledge, this represents the first report of the application of IVIS in a parasitic helminth.

## Materials and methods

### Construction of pBACII-BmGLuc-FLuc

pBACII-BmGluc-CHR [17] was digested with *Mlu* 1 to remove the CHR ORF. The plasmid backbone was purified on a 1% agarose gel and dephosphorylated using Antarctic Phosphatase. The firefly luciferase ORF was amplified from the pGL3-Basic vector (Promega, Madison, WI) using primers constructed with synthetic *Mlu*1 restriction sites on their 5' ends. The sequence of the primers were 5’ GGGACGCGTATGGAAGACGCCAAAAACATAAAGA 3’ and 5’ GGGACGCGTTTACACGGCGATCTTTCCGCCCTT 3’. The amplification reactions contained 1 μl *Pfu* Ultra II Fusion HS DNA Polymerase (Agilent, Santa Clara, California) and 5ng of pGL3-Basic DNA in a solution containing 1x *Pfu* Ultra II Fusion HS buffer (provided by the manufacturer), 0.2 μM of each primer and 250 μM of each dNTP. Cycling conditions consisted of an initial denaturation step of 2 minutes at 94 ^o^C, followed by 15 cycles consisting of 30 sec at 94 ^o^C, 30 sec at 55 ^o^C and 2 min at 72 ^o^C. The resulting product was cloned into the pCR2.1 cloning vector and its sequence confirmed. The firefly ORF was then digested from the pCR2.1 clone with *Mlu* 1, gel purified on a 1% agarose gel and ligated to the plasmid backbone prepared as described above. The sequence of the resulting clone was verified and the functionality of the luciferase markers further confirmed by biolistic transfection of *B*. *malayi* embryos as previously described [18].

### Lipofection of *B*. *malayi* L3

*B*. *malayi* molting larvae were transfected by lipofection, as previously described [17]. In brief, *B*. *malayi* infective larvae (L3) were obtained from the Filarial Research Reagent Repository (FR3). Prior to the arrival of the L3, individual wells of a 24-well tissue culture plate were seeded with Bovine Embryo Skeletal Muscle (BESM) cells (obtained from the NIH/NIAID Filariasis Research Reagent Resource Center). The cells were cultured for 1–2 days until they reached 70–90% confluency. Upon receipt, the L3 were washed five times with a solution consisting of RPMI 1640 medium containing 0.1x Antibiotic Antimycotic solution (Gibco) 10 μg/ml gentamycin and 2 μg/ml Ciprofloxacin. The L3 were then dispersed in 5 ml of CF-RPMI tissue culture medium [18]. The L3 were allowed to settle and all but 1ml of media removed. The media on the feeder cells was replaced with CF-RPMI and transwells (Costar) were placed in the wells. The dispersed L3 were then aliquoted among the transwells so that each transwell contained no more than an average of 100 L3. Additional CF-RPMI was added to the transwell to bring the total volume in the transwell to 500 μl. Transfections were initiated by diluting 24 μl Lipofectamine LTX reagent (Thermo Fisher) in 300 μl Opti-MEM medium (Thermo Fisher). A total of 3 μg of pBACII-BmGluc-FLuc plasmid DNA and 3 μg pBmCDTH plasmid DNA (encoding the *piggyBac* transposase driven by the *Bmhsp70* promoter) were then added to 300 μl Opti-MEM medium; 6 μl PLUS Reagent was then added to the DNA solution. The diluted DNA was then combined with 300 μl of the diluted Lipofectamine LTX Reagent to prepare the DNA micelles. The mixture was incubated for 5 minutes at room temperature. A total of 50 μl of the micelle solution was then added to each well containing the L3. The L3 were then cultured at 37°C under 5% CO_2_. The feeder cell medium was changed and additional lipofection complexes were added to the transwells containing the L3 on a daily basis. The medium in the transwells was not changed, so as not to disturb the larvae. On day 5, molting of the L3 was induced by the inclusion of ascorbic acid to a final concentration of 75 μM in the feeder cell medium. The L3 were incubated for a total of eight days. The transfected L3 were then collected and implanted into naive gerbils.

Male gerbils (*Meriones unguiculatus*) were obtained from Charles River (US), housed in in multiples of 2–3, and allowed to acclimate for one week prior to parasite implantation. Gerbils were infected subcutaneously (SQ) or intraperitoneally (IP) with approximately 100 transgenic *Brugia malayi* forth-stage larvae as previously described [17].

### IVIS visualization of parasites

Firefly Luciferase activity in infected gerbils was visualized using an intensified-charge-coupled device (I-CCD) video camera of the *in vivo* Imaging System (IVIS 100, Xenogen) [19]. Gerbils were anaesthetized using an isoflurane anesthesia system (XGI-8, Xenogen). Following this, gerbils were injected intraperitoneally with d-luciferin dissolved in PBS (100 mg/kg of body weight; Synchem, Kassel, Germany) and measurements were performed within 5 min of injection. Bio-luminescence imaging was acquired with an exposure time of 60 sec. Imaging data were analyzed using the living image (Xenogen) program.

Individual F1 microfilariae were collected from a gerbil infected with parasites transfected with pBACII-BmGLuc-FLuc and placed in individual wells of a 384 well plate with each well containing 25μl of CF-RPMI medium. A total of 25μl of a pre-warmed solution of 300 μg/ml d-luciferin in CF-RPMI was added to each well. The microfilariae were incubated at 37°C for 10 minutes and the plate imaged as described above. Following imaging, the plate containing the microfilariae was incubated overnight at 37°C under 5% CO_2_ and the medium assayed for GLuc, as previously described [17]

### Cryopreservation of transgenic larvae

The cryopreservation methods tested were modified from previously published methods for the cryopreservation of *B*. *malayi* microfilaria [20,21] and L3 [22]. In brief, freezing medium was prepared by mixing equal volumes of a Solution 1 (8mM Polyvinyl pyrrolidone (mw 40,000) and Solution II (20% FCS and 18% dimethyl sulfoxide in tissue culture medium). The mixture was filter sterilized and cooled on ice. Transgenic larvae were collected from the transwell cultures used for lipofection and added to 10 ml of Graces' insect medium at 4°C. The larvae were collected by centrifugation at 50 x g at 4°C for 5 minutes. The excess medium was removed from the larvae, leaving approximately 100 μl in the tube. The larvae in the remaining medium were transferred to a 2ml cryovial, and 1.7 ml of cold freezing solution was added. The larvae were then frozen using a CryoMed freezing apparatus (Fisher Thermo Scientific). The protocols tested were as follows:

- Grace’s Rapid Cool: Grace’s insect medium was used to prepare Solution II. The larvae were frozen at -1°C/min from 4°C to -4°C, followed by -10°C/min from-4°C to -90°C.
- Grace’s Slow Cool: Grace’s insect medium was used to prepare Solution II. The larvae were frozen at -0.8°C/min from 4°C to -4 C, followed by -1°C/min from -4°C to -90°C.
- MEM Slow Cool: MEM medium was used to prepare Solution II. The larvae were frozen at-0.8°C/min from 4°C to -4°C, followed by -1°C/min from -4°C to -90°C.

In all cases, the cryopreserved larvae were immediately transferred to liquid nitrogen, and stored for 14 days. The larvae were then removed and thawed in a 37°C water bath. The larvae were then transferred to 10ml of CF-RPMI medium at 37°C, subjected to centrifugation at 50 x g at room temperature for 5 minutes and all but 100ul of the medium discarded. The wash process was repeated once more, and the larvae transferred to transwells and co-cultured overnight with a BESM monolayer at 37°C under 5% CO_2_, as previously described [17]. In the morning, the larvae were collected from the transwells and implanted into the peritoneal cavity of naïve gerbils.

### Parasitological Evaluation of Animals infected with transgenic larvae

Peritoneal lavages were performed to obtain an estimate of the interperitoneal microfilarial burden of gerbils that had been interperitoneally infected with transgenic parasites at 131, 152, 166, 168, 200 and 253 days post-infection. Necroscopy was performed to assess parasite burden and organ involvement on 159, 166, 178, 195 and 200 days post-infection. Organs believed on the basis of the IVIS images to contain microfilaria were removed from the necroscopied animals, minced in tissue culture medium and incubated for 24 hours at 37°C under 5% CO_2_ to permit migration of microfilaria from the tissues. This was followed by microscopic examination of the organs and medium to detect the presence of parasites.

### Flubendazole treatment of infected gerbils

Flubendazole (FBZ) was obtained via Acros Organics (Thermo Fischer). FBZ was administered subcutaneously once a day at a dose of 10mg/kg for five consecutive days and the diluent for FBZ administration consisted of 0.5% Hydroxyethyl Cellulose, 0.1% Tween 80, and 99.4% sterile water in formulation consistent with previously documented treatment protocols [23]. Animals were imaged at 186 days post-infection, and treatments begun on that day. Post treatment IVIS imaging was carried out at 193 days post-infection (5 days after the start of treatment) and at 200 days post-infection (14 days after the start of treatment) to evaluate parasite clearance. Necroscopy was performed at 200 days post-infection followed by microscopic examination of the organs, as described above.

### Ethical statement

Protocols involving animals were reviewed by the Institutional Animal Care and Use Committee of the University of South Florida and were approved under protocol # R IS00003568. The Animal Facilities at the University of South Florida are fully accredited by AAALAC International as program #000434 and are managed in accordance with the Guide for the Care and Use of Laboratory Animals, the Animal Welfare Regulations, the PHS Policy, the FDA Good Laboratory Practices, and the IACUC Principles and Procedures of Animal Care and Use. The University of South Florida has assurance #D16-00589 (A4100-01) on file with OLAW/PHS and maintains registration #58-R-0015 with USDA/APHIS/AC.

## Results

**Fig 1:**
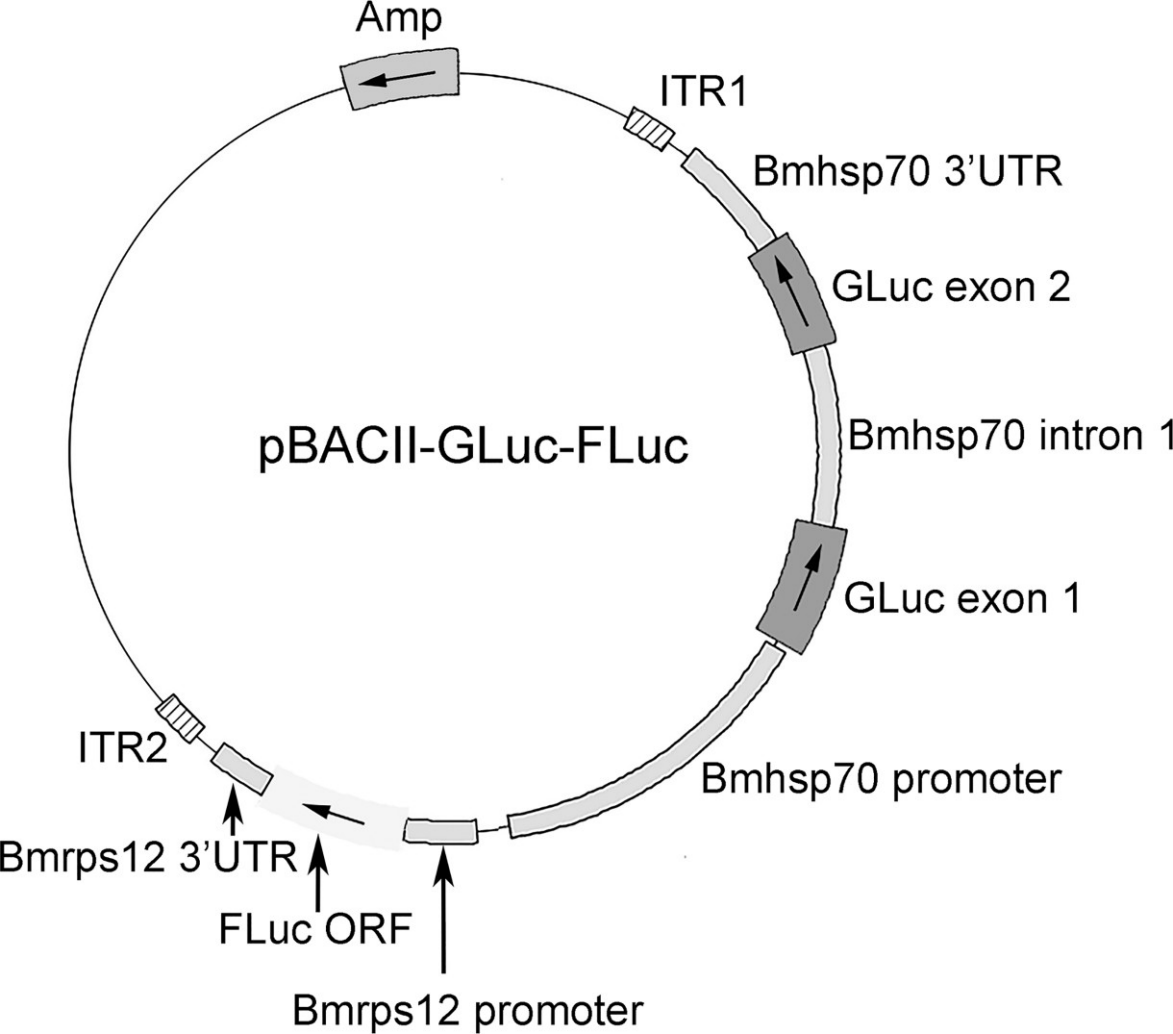
Feature map of pBACII-BmGLuc-FLuc: Amp = β lactamase antibiotic selection gene. ITR = *piggyBa*c inverted terminal repeats GLuc = *Gaussia princeps* luciferase ORF. FLuc ORF = firefly luciferase ORF. Arrows on the plasmid map indicate direction of transcription.

The dual luciferase plasmid pBACII-BmGLuc-FLuc was prepared by substituting the firefly luciferase (FLuc) open reading for the mCherry ORF in pBACII-BmGLuc-CHR [17]. pBACII-BmGLuc-FLuc contains the *piggyBac* transposon inverted terminal repeats (ITRs) flanking two expression cassettes (Fig 1).

The first of these consists of the *Gaussia princeps* luciferase (GLuc) ORF whose expression is driven by the *Bmhsp70* promoter and *Bmhsp70* 3’ UTR. The first intron of the *Bmhsp70* gene was inserted into the GLuc ORF, which provides the sequences necessary and sufficient to direct trans splicing of the mRNA produced from transcription of the expression cassette. As GLuc is secreted from transfected *B*. *malayi* it can be used to identify transgenic parasites in a non-invasive manner [17]. The second expression cassette consists of the FLuc ORF driven by the *Bmrps12* promoter and 3’ UTR. Both the *Bmhsp70* and *Bmrps12* genes are expressed in all lifecycle stages of *B*. *malayi* [24].

**Fig 2:**
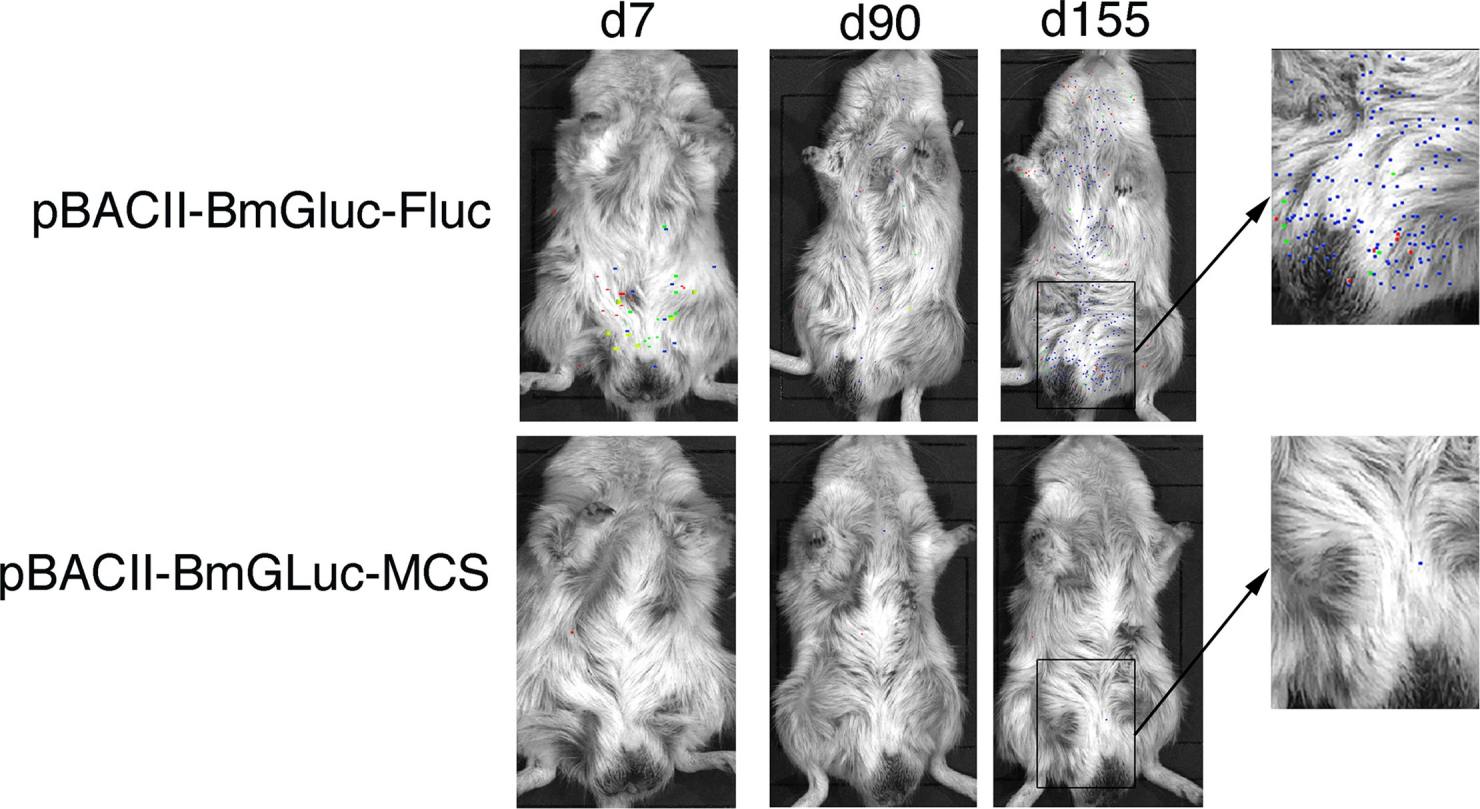
IVIS Visualization of transgenic parasites in infected gerbils: Gerbils were infected by IP injection with transgenic parasites and the animals imaged periodically beginning at 7 days through 155 days post-infection, as described in Materials and Methods. Colors represent the relative level of luciferase activity ranging from low (blue), to medium (green), to high (yellow, red). pBACII-BmGLuc-FLuc = animal infected with parasites transfected with pBACII-BmGLuc-FLuc plus pBmCDTH and pBACII-BmGLuc-MCS = animal infected with parasites transfected with pBACII-BmGLuc-MCS plus pBmCDTH. Arrows point to images of the boxed area on the abdomens of the animals at 155 days post-infection that have been enlarged for clarity. The images shown are representatives taken from five separate experiments that were carried out overall. Each experiment consisted of between 2 and 3 animals which were infected with parasites transfected with pBACII-BmGLuc-FLuc and pBmCDTH, together with control animals infected contemporaneously, either with parasites transfected with pBACII-BmGLuc-MCS plus pBmCDTH (shown here) or untransfected parasites (e.g. Fig 3).

**Fig 3:**
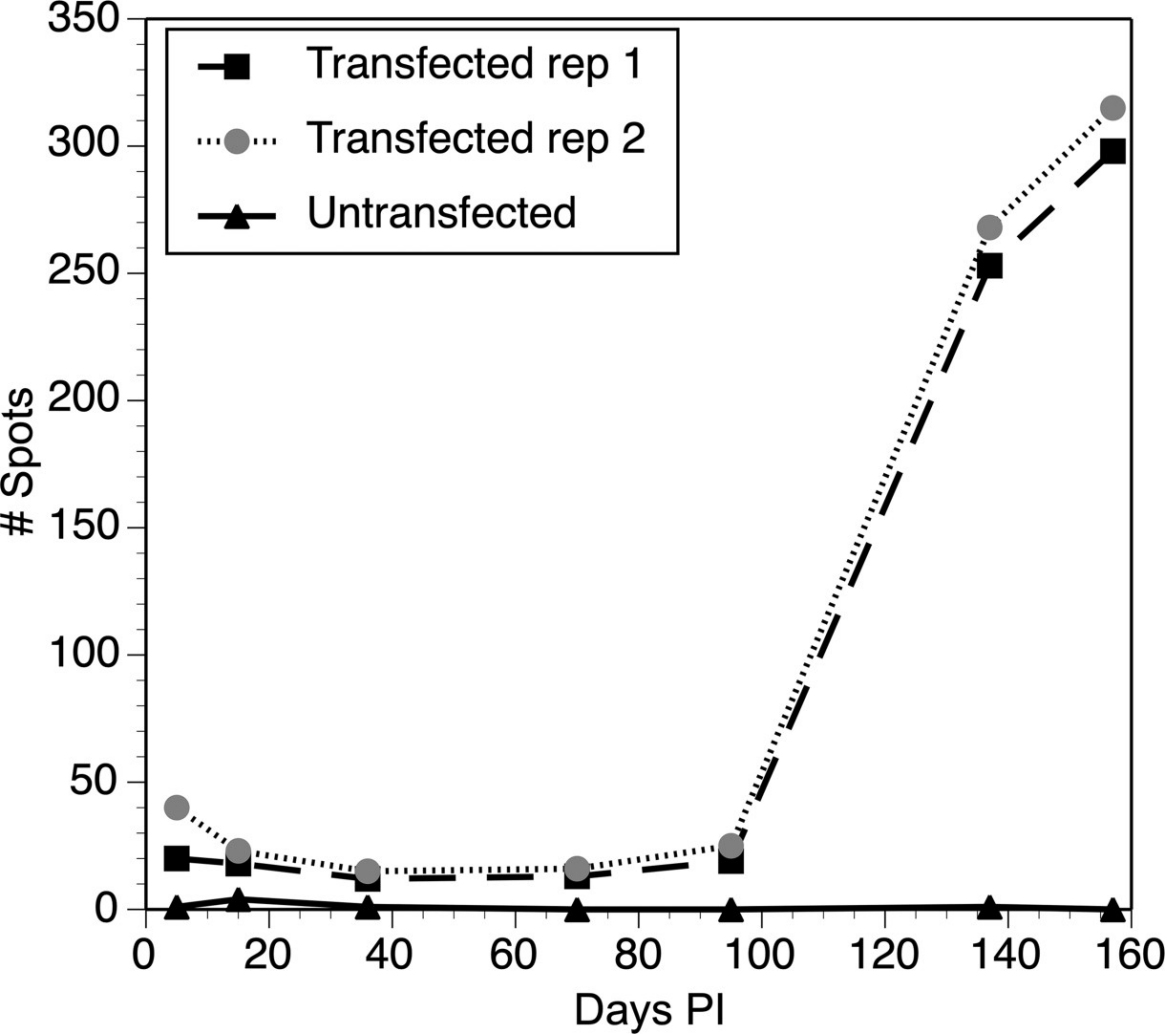
Quantification of IVIS signals in animals infected with transgenic parasites: Values represent total photon counts in each animal imaged. Transfected rep 1 and Trasnfected rep2 = results from two different animals infected with parasites transfected with pBACII-BmGLuc-FLuc and pBmCDTH. Untransfected = results from a contemporaneous control animal infected with untransfected parasites. The graph shown is a representative experiment taken from five separate experiments that were carried out overall. Each experiment consisted of between 2 and 3 animals which were infected with parasites transfected with pBACII-BmGLuc-FLuc and pBmCDTH, together with control animals infected contemporaneously, either with untransfected parasites (shown here) or with parasites transfected with pBACII-BmGLuc-MCS plus pBmCDTH (e.g. Fig 2).

pBACII-BmGLuc-FLuc together with pBmCDTH (which encodes the *piggyBac* transposase driven by the *Bmhsp70* promoter) was used to transfect *B*. *malayi* infective stage larvae (L3) by lipofection in a co-culture system with Bovine Embryo Skeletal Muscle (BESM) cells, as previously described [17]. After the cultured parasites had completed the L3-L4 molt, 100 transgenic parasites were implanted into naïve gerbils. The gerbils were then monitored periodically using the *in vivo* imaging System (IVIS). Example IVIS images are presented in Fig 2 and typical results summarized graphically in Fig 3.

**Fig 4:**
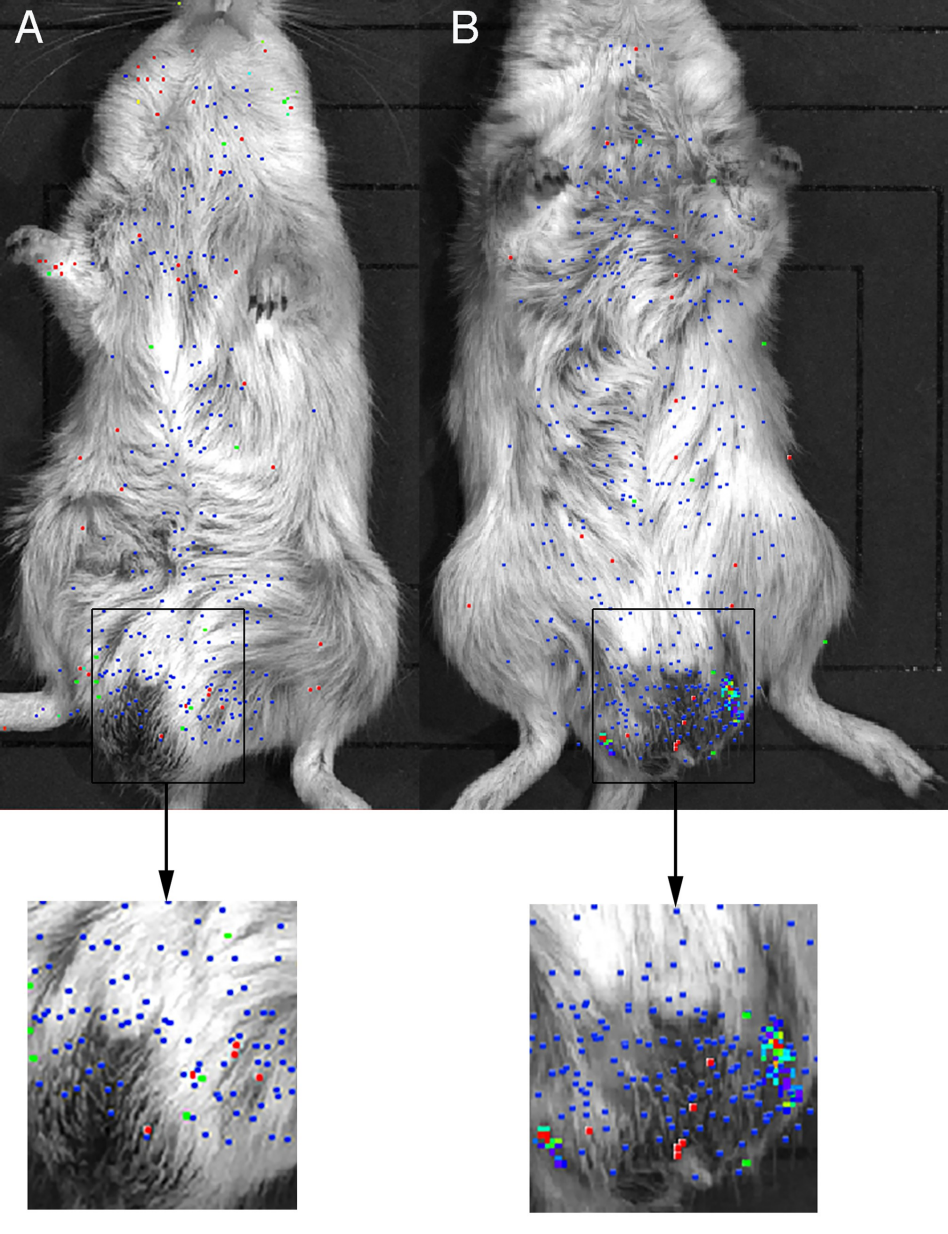
IVIS visualization of animals infected IP or SQ with parasites transfected with pBACII-BmGLuc-FLuc and pBmCDTH. Panel A: Animal infected IP with parasites transfected with pBACII-BmGLuc-FLuc and pBmCDTH. Panel B: Animal infected SQ with parasites transfected with pBACII-BmGLuc-FLuc and pBmCDTH. Arrows point to the boxed areas on the full animal images that have been enlarged for clarity.

IVIS detected signals at 7 days post-infection through day 100 in animals infected with parasites transfected with pBACII-BmGLuc-FLuc and pBmCDTH (Fig 2). In contrast, little or no signal was detected in animals infected with parasites transfected with pBACII-BmGLuc-MCS (the plasmid backbone used to create pBACII-BmGLuc-FLuc, which lacks the FLuc ORF) and pBmCDTH (Fig 2). This difference was statistically significant (mean number of spots detected in this period in animals infected with parasites transfected with pBACII-BmGLuc-FLuc and pBmCDTH = 31; mean number of spots detected in this period in animals infected with parasites transfected with pBACII-BmGLuc-MCS and pBmCDTH = 1; p < 0.001, t test). The signal decreased gradually in the animals infected with parasites transfected with pBACII-BmGLuc-FLuc and pBmCDTH through 36 days post infection, at which the signal seemed to stabilize until roughly 100 days post infection, when the parasites would be expected to have begun to reach maturity and to have begun producing progeny microfilaria (Fig 3). At that point, the signal in the pBACII-BmGLuc-FLuc and pBmCDTH parasite infected animals increased significantly (p < 0.01; t test), presumably reflecting the production of transgenic progeny microfilaria (Figs 2 and 3). In contrast, no signal increase was seen in the animals transfected either with parasites transfected with pBACII-BmGLuc-MCS and pBmCDTH (Fig 2) or in animals infected with untransfected parasites (Fig 3). Late in the infection, the signals were distributed fairly evenly throughout the body of IP infected gerbils, while the density of the signal appeared highest in area of the testis of the SQ infected animals (Fig 4).

Peritoneal lavages were performed to obtain an estimate of the microfilarial burden in gerbils that had been interperitoneally infected with transgenic parasites at 131, 152, 166, 168, 200, and 253 days post-infection. No microfilaria were detected in the lavage fluid through 200 days post-infection. However, numerous microfilariae were detected in the lavage fluid at 253 days post-infection. Necroscopy was performed to determine parasite burden and organ involvement on some of the animals interperitoneally infected with the transgenic parasites on 159, 166, 178, 195, and 200 days post-infection, as described in Materials and Methods. Large numbers of microfilariae were detected in the testes and lymph nodes in all gerbils infected with the transgenic parasites as early as the first harvest on day 159 post-infection.

**Fig 5:**
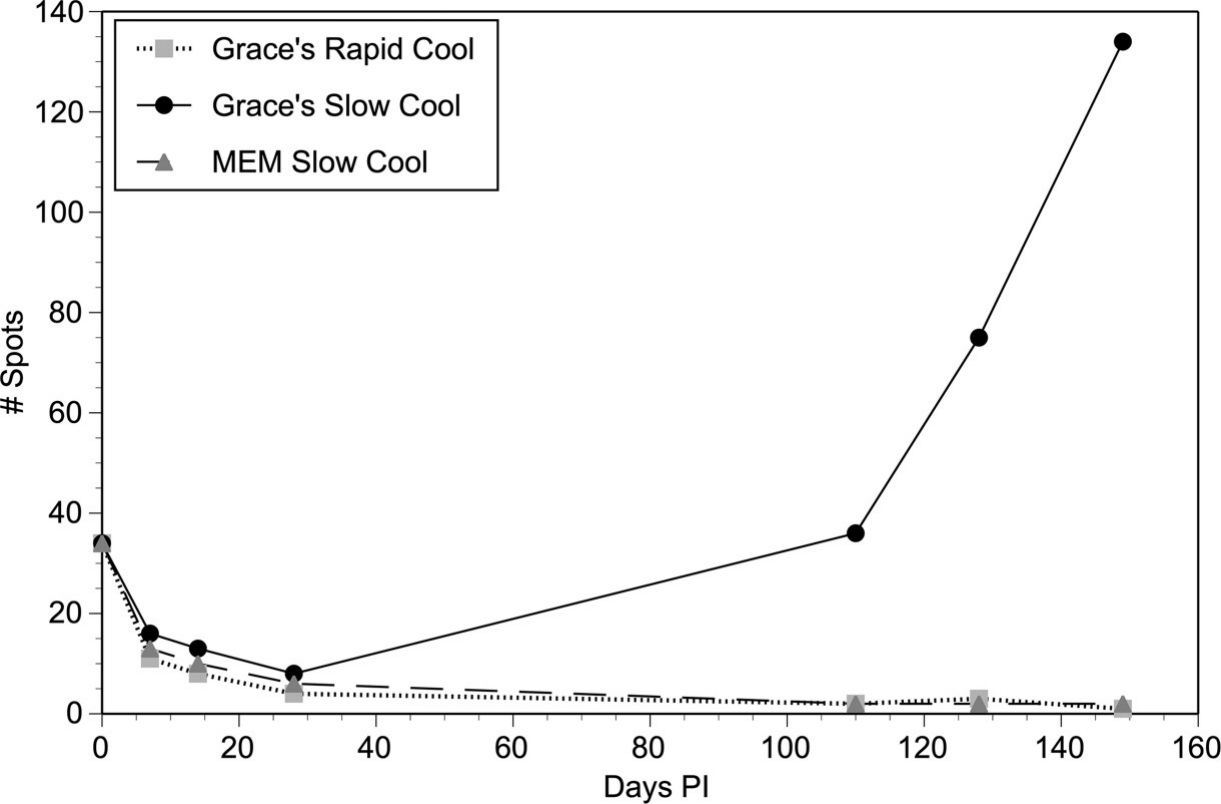
Quantification of IVIS signals in animals infected with transgenic parasites cryopreserved with different methods. Parasites were transfected with pBACII-BmGLuc-FLuc and pBmCDTH and cryopreserved with three different methods as described in Materials and Methods. Values represent total photon counts in each animal imaged. The results shown are an example from two independent experiments.

The *piggyBac* system results in the integration of the transgenes into the genome of the parasite, making it possible to create stable integrated transgenic parasite lines [17]. However, in order to maintain such lines over the long term, a method to cryopreserve the transgenic parasites would be highly desirable. In developing such a cryopreservation method, we felt that it would be best to target the larval stages, as these could be thawed and directly implanted into gerbils, bypassing the need for laboratories to maintain an insectary. To develop a cryopreservation method suitable for transgenic larvae, three protocols for cryopreservation described in Materials and Methods were evaluated using infected larvae transfected with pBACII-BmGLuc-FLuc and pBmCDTH. The transfected larvae were cryopreserved following the different protocols, stored for 2 weeks in liquid nitrogen, thawed and injected into naïve gerbils. The viability and developmental competence of the parasites was then assessed by IVIS imaging of the infected animals. At the start of the experiment, the signal from all the animals injected with the parasites cryopreserved using the different methods were similar (Fig 5).

The signals declined in all the animals through day 28. However, when examined 110 days post infection the signal had continued to decline in the animals injected with parasites preserved using the Grace’s Rapid Cool and MEM Slow Cool protocols, while the signal dramatically increased in the animal injected with the parasites cryopreserved using the Grace’s Slow Cool protocol (Fig 5).

**Fig 6:**
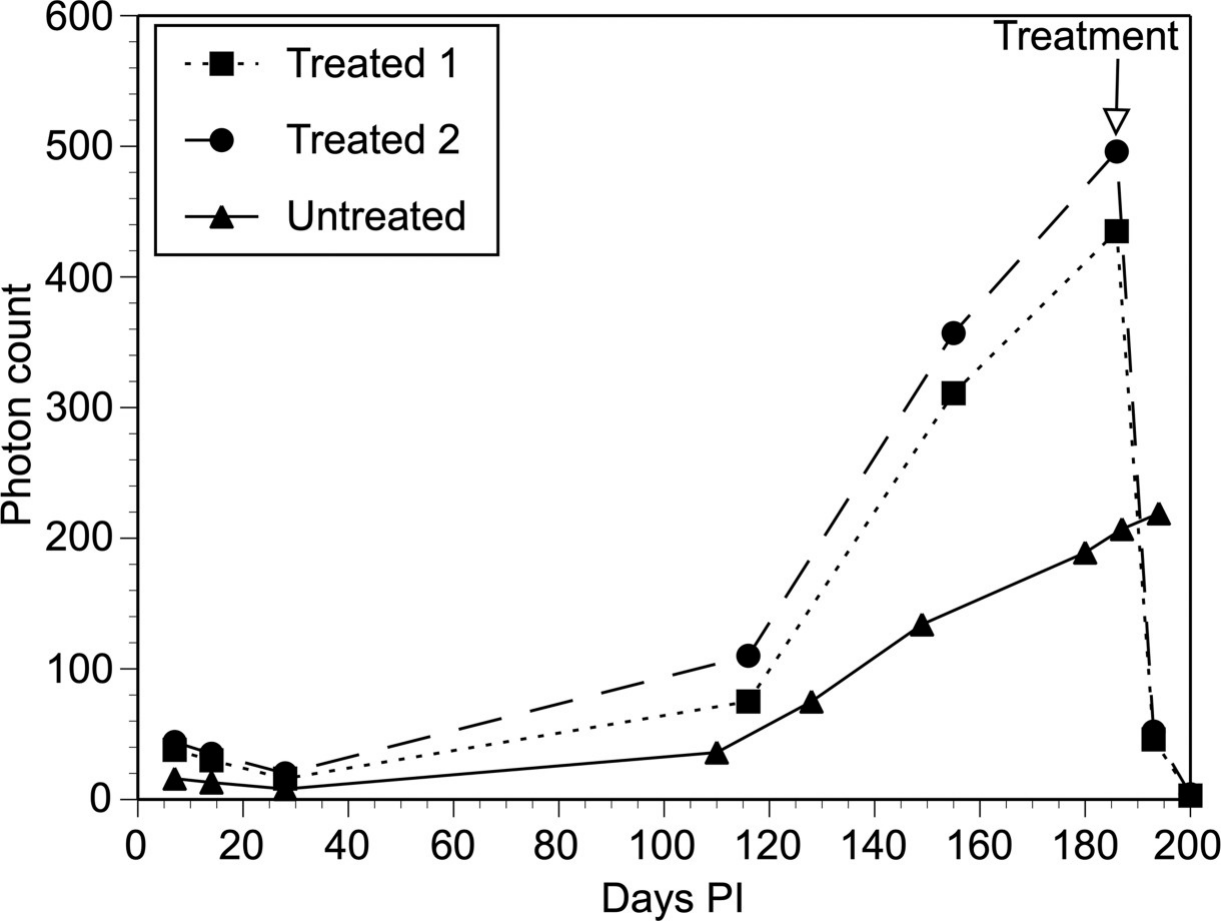
Quantification of IVIS signals in animals infected with transgenic parasites and treated with flubendazole. Parasites were transfected with pBACII-BmGLuc-FLuc and pBmCDTH and the animals labeled “Treated” (n = 2) were treated with flubendazole as described in Materials and Methods. The animal labeled “Untreated” (n = 1) was a contemporaneous gerbil infected with parasites transfected with pBACII-BmGLuc-FLuc and pBmCDTH, but not treated with flubendazole. Values represent total photon counts in each animal imaged.

One potential application of using IVIS to detect *B*. *malayi* could be as an assay to non-invasively monitor the effect of various drugs on parasites *in vivo*. As an initial test of the potential utility of this application, three gerbils were implanted with parasites transfected with pBACII-BmGLuc-FLuc and pBmCDTH and allowed to develop for 186 days. Two gerbils were then treated with flubendazole (a potent antifilarial drug [25]) as described in Materials and Methods, and the effect of treatment monitored by IVIS. The parasite burden in the infected animals was extremely high prior to treatment (Fig 6).

Following treatment, the IVIS signal declined to close to zero in the treated animals, while the signal continued to increase in the untreated animal (Fig 6). This suggested that the decline in the IVIS signal was documenting the death of the parasites in the treated animals. To confirm this hypothesis, the treated animals were euthanized and subjected to necroscopy. As expected, no viable parasites were found in the treated animals.

**Fig 7:**
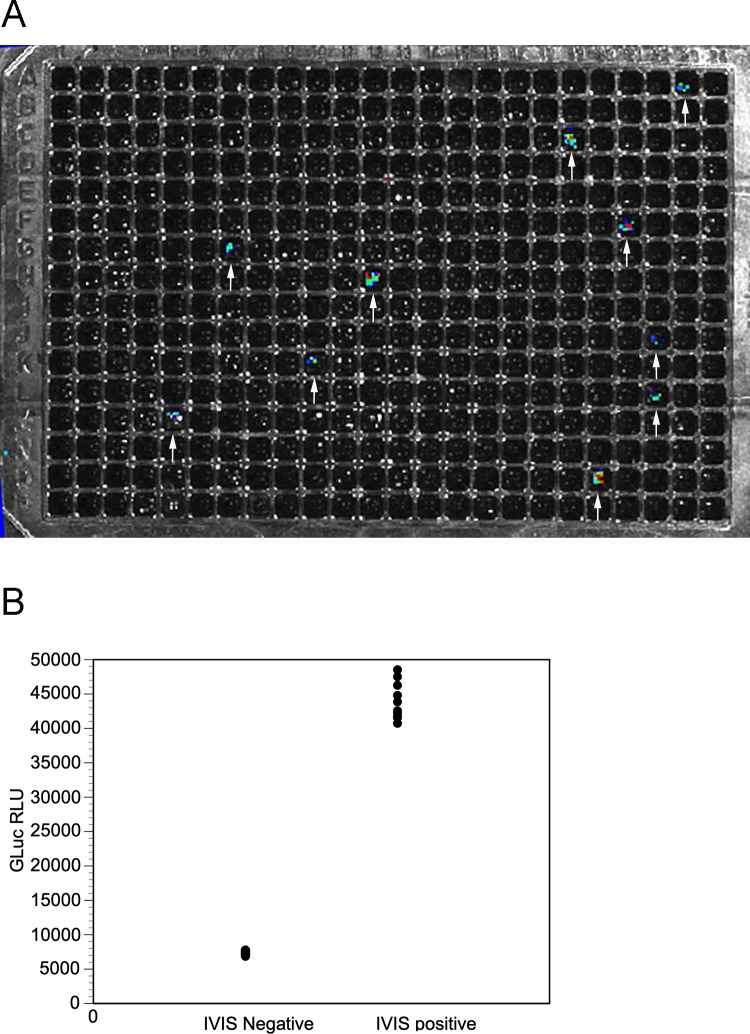
IVIS visualization of individual first-generation microfilariae from an animal infected with parasites transfected with pBACII-BmGLuc-FLuc and pBmCDH. Panel A: IVIS visualization of a 384 well plate with each well containing an individual microfilariae. Arrows highlight positive wells. Panel B: GLuc activity in wells identified as negative (n = 374) or positive (n = 10) by IVIS.

Given that the IVIS signal increased dramatically beginning at around 100 days post infection when the larvae implanted into the animals were expected to mature and begin producing progeny, we hypothesized that the individual spots we were detecting in the IVIS corresponded to individual F1 transgenic microfilariae. To test this hypothesis, we isolated microfilaria from an animal 150 days post infection that had been implanted with parasites transfected with pBACII-BmGLuc-FLuc and pBmCDTH and placed individual microfilariae into separate wells of a 384 well plate. d-Luciferin was added to the plate and the plate imaged in the IVIS apparatus. A total of 10 wells were found to produce a detectable signal in the IVIS (Fig 7, Panel A).

The plate was then incubated overnight at 37°C and the medium in all wells assayed for GLuc activity (the second reporter in pBACII-BmGLuc-FLuc). All of the wells producing a detectable signal in the IVIS image contained GLuc activity which was roughly five fold greater than that seen in the wells which did not have a detectable IVIS signal (p < 0.0001; t test; Fig 7, Panel B). In contrast, none of the microfilaria in the wells that did not have a detectable signal in the IVIS secreted GLuc into the medium.

## Discussion

To our knowledge, this represents the first report of the use of IVIS to monitor infection of an animal model with a parasitic helminth. The ability to non-invasively monitor the progress of a filarial infection will be useful for a number of different research applications. First, as we have demonstrated in principal in the studies reported here, animals infected *B*. *malayi* transfected with pBACII-BmGLuc-FLuc can provide a rapid and non-destructive way to evaluate the efficacy of potential new chemotherapeutic agents *in vivo*. Firefly luciferase is particularly useful in this regard, as luciferase requires ATP as a substrate. ATP is a very labile molecule, and the cellular stores of ATP decline rapidly after death, making ATP a rapidly responding marker of viability [26]. This has obvious advantages when one is attempting to evaluate the anti-filarial activity of new chemotherapeutic agents. Similarly, it is possible to envision using this system to study the mechanism and efficacy of potential vaccines against the filaria, as has been done in studies of malaria [13].

Another potential use of this model could be to study tissue tropism and parasite development in infected animals. Previous such studies in filaria have required careful examination of isolated tissues from infected animals to locate parasites [27]. This is both laborious and destructive, so the course of the infection cannot be monitored repeatedly in a single infected animal. The use of IVIS to detect transgenic parasites will allow parasites to be rapidly and non-destructively located in an infected animal, and a single animal can be re-imaged repeatedly, providing one with a time course of parasite movement and development.

The experiments above also demonstrate that IVIS can be used to identify individual transgenic parasites expressing FLuc. It is possible to envision using the IVIS as a selection for transgenic parasites expressing the FLuc reporter. This method has some advantages over selection of transgenics by assaying the medium of cultured parasites for secreted GLuc. The IVIS based assay can be performed in less than an hour, eliminating the need for overnight culture of the parasites, which is required to detect secreted GLuc activity [17].

In previous studies using biolistic transfection of isolated *B*. *malayi* embryos, a dual luciferase assay has been used to identify essential elements [28] [29] and regulatory regions [30,31] of *B*. *malayi* promoters. However, as these studies employed isolated embryos which were not developmentally competent, the dual luciferase approach could not be applied to study developmental regulation of genes expressed at particular life cycle stages. As pBACII-BmGLuc-FLuc carries two luciferase reporters it is likely that this construct (when modified to drive the expression of one of the luciferase reporters from putatively developmentally regulated promoters) will be useful in exploring the cis acting factors involved in stage specific gene expression in *B*. *malayi*. However, given the semi-random nature of the insertion sites targeted by the *piggyBac* system (TTAA) such studies might be complicated by position effects resulting from integration of individual constructs into different sites in the genome. Such position effects may explain the variations in signal intensity we observed in the IVIS images (c.f. Figs 3 and 4). This might be controlled for by using one of the luciferase reporters as an internal normalization control; however developing a method that permits precise integration into a single site in the genome would overcome this problem altogether. Clustered Regularly Interspaced Short Palindromic Repeats (CRISPR) based technology [32], which permits precise targeting of sequences in the genome, would provide a solution to this issue. Efforts are currently underway to adapt CRISPR technology to *B*. *malayi*.

pBACII-BmGLuc-FLuc was constructed with a backbone containing the *piggyBac* transposon inverted terminal repeats (ITRs) flanking the two Luc expression cassettes. Plasmids with this backbone, when co-transfected with pBmCDTH, a plasmid encoding the *piggyBac* transpose under the control of the *Bmhsp70* promoter, are stably integrated into TTAA sites in the parasite genome [17]. Thus, by selecting transgenic F1 parasites, it should be possible to create a stable transgenic parasite line containing the dual luciferase expression cassettes integrated into the parasite genome. With the development of a method to cryopreserve transgenic larvae, it should be possible to preserve the line over the long term and to make this line available to any interested investigator. Work to produce such a line is currently underway.
